# Ophthalmic Manifestations of Syphilis

**Published:** 1909-02-13

**Authors:** 


### Ophthalmology.

### OPHTHALMIC MANIFESTATIONS OF SYPHILIS.

### I. EXTRA-OCULAR.

Syphilitic disease manifests itself in and around the eye in many and very varied forms, some readily recognisable, others most obscure, some giving rise to slight damage, others not only to total loss of vision but to complete destruction and shrinking (phthisis bulbi) of the globe itself. The conjunctiva may be the seat of the original infection (chancre of the lid); the eye may suffer throughout the secondary and tertiary stages; and there are also eye conditions generally conceded to be due to congenital syphilis. Any portion of the eye may become the seat of syphilitic lesions with the single exception of the lens, and even this is indirectly affected by reason of the involvement of the uveal tract and consequent lack of nourishment.

The bony walls of the orbit may be the seat of syphilitic periostitis, and this may be one of the first signs of constitutional infection, closely following the initial sore. Orbital periostitis gives rise to great pain around the eye, with protrusion of the globe itself, swelling and redness of the eyelids, with injection and sometimes chemosis of the conjunctiva. The superior margin of the orbit becomes very tender to pressure, although the inflammation may be deeply seated and not involve the margin at all. The pain invariably becomes worse at night and the protrusion of the eyeball worse at the same time. The optic nerve may be pressed upon and optic neuritis may lead to atrophy of the nerve and loss of sight. If an abscess form it may point through the conjunctiva, in which case it indicates that the bone lesion is deep in the orbit; or at the orbital margin, that is through the lid, thus indicating a lesion at, or close to, the bony margin of the orbit outside the attachment of the orbital fascia. The eyeball is protruded towards one side or the other, rarely straight forwards, because one or the other wall of the orbit is more affected than the other. Such cases do not often go as far as suppuration, but react to vigorous treatment. Long continued periostitis of one of the bones forming the orbit may lead to an exostosis, or to several which may ultimately coalesce to form one large protrusion; such exostoses usually spring from the upper and inner walls of the orbit, the os planum of the ethmoid being a very favourite region. They are frequently a late manifestation of syphilis.

Stricture of the lachrymal duct is also caused by extension of gummatous inflammations from both the conjunctival sac and the mucous membrane of the nose. Again, a very large number of bony strictures of the nasal duct are directly caused by syphilitic periostitis or necrosis of the lachrymal bone and surrounding bones. Gummata have been known in both the lachrymal sac and duct; on breaking down they may cause dacryocystitis of a very severe and obstinate type. The chronic specific inflammations of the nasal mucous membrane so frequently met with in congenital syphilis cause inflammation of the lachrymal duct and sac by extension, and are very difficult to treat short of removing the sac altogether.

Inflammation of the sclerotic coat of the eyeball is not infrequently met with. It may take the form known as episcleritis, in which the inflammation is circumscribed and affects only a small area of the sclera, that part covered by the conjunctiva; or it may affect, more or less, the whole of the anterior part of the eyeball. In the latter case it most usually accompanies cyclitis, and the circle round the cornea corresponding to the site of the ciliary body becomes very red, deeply congested, and painful on pressure. The conjunctiva over it becomes œdematous and the sclerotic itself becomes thickened, and at times raised into circular swellings; these later on begin to look bluish in colour and bulge outwards, showing that the sclera has thinned and given way before the intra-ocular tension. In this manner the large anterior staphylomata are formed which are seen in eyes affected with syphilis, either congenital or acquired.

The tarsal cartilage, usually that of the upper lid, at times becomes much thickened, the skin covering it chronically reddened, and the conjunctiva lining it hypertrophied and congested; this has been called tarsitis, and an inflammation originating thus in the tarsus itself is invariably specific.

The conjunctiva itself is occasionally the seat of the primary sore, which is most frequently close to the margin of the lid or in the retrotarsal folds, and is surrounded by the characteristic induration found in other mucous tracts when similarly affected. At times this induration may not be present, and there is simply a soft ulcerated patch more difficult to diagnose correctly. The pre-auricular and submaxillary glands are invariably affected.

The cornea is affected by hereditary syphilis more frequently and to a greater extent than by the acquired affection. It is often involved secondarily, that is to say choroiditis may be present for some time before any affection of the cornea shows itself. Or obstinate inflammation of the ciliary body may be of some weeks standing before the cornea begins to look cloudy. This haze increases and in a very short time a typical parenchymatous, or interstitial, keratitis manifests itself. This deeply seated inflammation of the cornea is invariably accompanied by other manifestations of hereditary syphilis, of which the commonest are a sunken nasal bridge, ozœna, enlarged glands in the neck which are hard and quite painless, Hutchinson's notched teeth, scars at the angles of the mouth, scars in the palate the result of previous ulcerations, and progressive deafness. Also there may be periosteal swellings along the shafts of the long bones, the anterior border of the tibiæ being usually affected. This very tedious inflammation of the cornea affects children between the ages of six and twenty, females more often than males; as a rule it attacks both eyes, not simultaneously but at a considerable interval of time. The very mildest case probably continues three months; and bad cases are as many years before the cornea regains transparency. The course of the disease may be divided into two stages: first, the active inflammatory stage in which distinct irritative symptoms are present; second, the clearing-up stage in which symptoms of irritation are but slight, and the diminution of the corneal opacity very gradual. A frequent painful complication is a tendency to atropine irritation; this is most unfortunate, for atropine is absolutely necessary for its sedative and mydriatic action.

